# X-ray fluorescence analysis of iron and manganese distribution in primary dopaminergic neurons

**DOI:** 10.1111/jnc.12073

**Published:** 2012-12-05

**Authors:** Tanja Dučić, Elisabeth Barski, Murielle Salome, Jan C Koch, Mathias Bähr, Paul Lingor

**Affiliations:** * The HASYLAB – DESYHamburg, Germany; † Department of Neurology, University Medicine GöttingenGöttingen, Germany; ‡ European Synchrotron Radiation Facility (ESRF)Grenoble, France; § Cluster of Excellence “Nanoscale Microscopy and Molecular Physiology of the Brain”Göttingen, Germany

**Keywords:** iron, manganese, oxidative state, Parkinson's disease, primary dopaminergic neurons, X-ray fluorescence microscopy

## Abstract

Transition metals have been suggested to play a pivotal role in the pathogenesis of Parkinson's disease. X-ray microscopy combined with a cryogenic setup is a powerful method for elemental imaging in low concentrations and high resolution in intact cells, eliminating the need for fixation and sectioning of the specimen. Here, we performed an elemental distribution analysis in cultured primary midbrain neurons with a step size in the order of 300 nm and ∼ 0.1 ppm sensitivity under cryo conditions by using X-ray fluorescence microscopy. We report the elemental mappings on the subcellular level in primary mouse dopaminergic (DAergic) and non-DAergic neurons after treatment with transition metals. Application of Fe^2+^ resulted in largely extracellular accumulation of iron without preference for the neuronal transmitter subtype. A quantification of different Fe oxidation states was performed using X-ray absorption near edge structure analysis. After treatment with Mn^2+^, a cytoplasmic/paranuclear localization of Mn was observed preferentially in DAergic neurons, while no prominent signal was detectable after Mn^3+^ treatment. Immunocytochemical analysis correlated the preferential Mn uptake to increased expression of voltage-gated calcium channels in DAergic neurons. We discuss the implications of this differential elemental distribution for the selective vulnerability of DAergic neurons and Parkinson's disease pathogenesis.

Parkinson's disease (PD) is the most frequent neurodegenerative movement disorder with a prevalence of about 1% in a population older than 70 years and about 3% in individuals older than 80 years (Strickland and Bertoni 2004). Pathological hallmarks include, but are not confined to, the preferential loss of dopaminergic (DAergic) neurons within the substantia nigra pars compacta and the presence of intracytoplasmic inclusions containing α-synuclein and ubiquitin, so-called Lewy bodies (Braak *et al*. 2003).

Next to the generation of free radicals in the course of dopamine metabolism, the increased presence of iron in the midbrain of PD patients has been assumed as a contributing pathogenic factor (Berg and Hochstrasser 2006). Iron participates in metabolic processes by undergoing oxidation–reduction reactions, a common property among transition metals, which allows this metal to undergo interconversion between the divalent cationic or ferrous (Fe^2+^), and trivalent cationic or ferric (Fe^3+^) states. These electron exchange processes can also lead to significant oxidative damage via free radical production within the brain when excess iron is present. In DAergic neurons during the dopamine catabolism, hydrogen peroxide is produced that in presence of iron favors Fenton reactions and oxidative stress [reviewed in (Papanikolaou and Pantopoulos 2005)].

Other transition metals such as Mn or Zn have been suggested to contribute to the pathogenesis of PD. For example, exposure of workers to high concentrations of manganese is an established risk factor for the development of a Parkinsonian syndrome, which clinically greatly mimics idiopathic Parkinson's disease [reviewed in (Guilarte 2010)]. Similar symptoms have been observed in psychostimulant drug abusers after repetitive intravenous injection of manganese-containing substances (Sikk 2011). Excessive levels of brain manganese have been linked to the loss of dopamine in the striatum, death of non-DAergic neurons in the globus pallidus, and damage of other neuronal pathways such as glutamatergic and GABAergic projections, all of which contribute to altered behavior, motor dysfunction, and cognition deficit (Erikson 2002). Manganese elevates intracellular H_2_O_2_ and related peroxides (HaMai *et al*. 2001) and reduces tyrosine hydroxylase activity and intracellular antioxidant levels (GSH, thiols, catalase) in DAergic neurons (Migheli 1999).

Iron and manganese are known to pass across the blood–brain barrier [reviewed in (Yokel 2009; Moos 2007)]. Because of their chemical similarity, both metals share and compete for transport proteins in organisms ranging from bacteria to mammals. As such, during conditions of low iron, abnormal manganese accumulation occurs. Conversely, when manganese concentrations are altered, the homeostasis and deposition of iron and other transition metals are disrupted. However, the knowledge on the subcellular allocation of trace elements upon exposure to manganese or iron remains incomplete. Indirect methods, like radioactive ^54^Mn tracing after cellular extraction, are able to quantify manganese in cell extracts, but provide only limited spatial information (Kalia *et al*. 2008).

The principle of X-ray fluorescence (XRF) is based on the irradiation of sample atoms with X-rays with energy sufficient to eject an electron from one of the atom's inner shells. The relaxation process is accompanied by the emission of a fluorescence photon. The energy of the emitted X-rays is characteristic of the excited element enabling the identification of the composition of the atoms constitutive of the sample. Beside element composition, X-ray spectromicroscopy performed using an X-ray nanoprobe beam is a unique method to determine the oxidation state of elements in cells and tissues (Qin 2011). X-rays can penetrate thick cells and tissues, eliminating the need of invasive preparation and sectioning of the specimen. Several studies have thus recently demonstrated the use of X-rays for trace element mapping of different cell types (Ortega 2007; Bacquart 2007; Ducić 2011).

This study was designed to investigate the subcellular distribution of manganese and iron in primary midbrain neurons and to compare this distribution in the DAergic and non-DAergic subpopulations by X-ray fluorescence microscopy. Previous studies have been limited to cell lines, post-mortem tissue, or did not take into account changes in the oxidative state during sample processing (Bacquart 2007; Szczerbowska-Boruchowska 2007). In this study, we visualized the transition metal distribution upon exposure in primary midbrain neurons and used the cryo-preserving (so-called cryo-embedding) technique to preserve oxidative states of the metal ions, which, to the best of our knowledge, is the first description exploiting primary midbrain neurons culture model with X-ray imaging techniques.

## Materials and methods

### Primary midbrain dopaminergic neuron culture

Our study design aimed at the characterization of differential characteristics of dopaminergic versus non-DAergic neurons and therefore required a reliable identification of the DAergic subpopulation in the living culture *in vitro*. To this means, we used a transgenic mouse line expressing green fluorescent protein (GFP) under the tyrosine hydroxylase (TH) promoter (TH–GFP mice). This results in the expression of GFP in ∼ 90% of all DAergic neurons in the substantia nigra pars compacta, as compared to immunohistochemical identification (Matsushita 2002). TH is the rate-limiting enzyme of dopamine synthesis and a marker for DAergic neurons in the midbrain.

The mesencephalon floor of embryonic day 12.5 TH–GFP mice (bred in the central animal facility of Göttingen University) was dissected and dissociated for a primary cell culture as previously described (Lingor *et al*. 2000; Shimoda 1992). Briefly, the brainstem was isolated and the meninges were removed. A medial incision was made at the ventricular opening of the tectum. The rostral parts of the ventral brainstem and of the tectum were cut away. The dissected tissue pieces were collected in ice-cold calcium–magnesium-free medium and centrifuged at 1.5 *g* for 4 min. Trypsin (750 μL, 0.25%, Sigma, Taufkirchen, Germany) was added to the tissue pellet, and after 15 min of incubation at 37°C was inactivated with 750 μL cold fetal calf serum. Dissociation of tissue fragments was achieved by gentle trituration using a fire-polished Pasteur pipette. The cell suspension was centrifuged at 73 *g* for 4 min and resuspended in culture medium.

Cells were seeded on silicon nitride membranes (1.5 × 1.5 × 2 · 10^−4^ mm, Silson, UK) and maintained for 2 days at 37°C in a 5% CO_2_-humified atmosphere in Dulbecco's modified eagle medium-F12 (Invitrogen, Scotland, UK) supplemented with 2.5 mg/mL bovine serum albumin (35%), 0.9% D-(+)-glucose solution (45%), 2 mM l-glutamine (PAA Laboratories, Pasching, Austria), 5 μg/mL insulin, 1 : 100 N1 medium supplement, and 1 : 100 PSN antibiotic mixture (Invitrogen). Medium was changed 24 h after cell dissection and subsequently every second day.

This midbrain culture contained ∼5–10% DAergic neurons and ∼ 90–95% GABAergic neurons with only single glial fibrillary acidic protein-positive cells being detectable throughout the culture time (Lingor *et al*. 2000). DAergic neurons could be easily identified *in vitro* by conventional inverted fluorescence microscopy using an EGFP filter.

### Immunocytochemistry and quantification of VGCC expression

Midbrain dopaminergic neuron (MDN) cultures were prepared as described above. At day *in vitro* 2 (DIV 2) or DIV 5 cultures were fixed in paraformaldehyde 4% for 10 min at 22°C, permeabilized with 100% ice-cold acetone (AppliChem, Darmstadt, Germany) for 10 min at −20°C, washed twice with phosphate-buffered saline and blocked with Antibody Diluent (Dako, Hamburg, Germany) for 10 min at 20°C. Probes were incubated with the primary antibodies (anti-tyrosine hydroxylase [T1299]; 1 : 500; Sigma) and anti-rat skeletal muscle voltage-gated calcium channel (α1 subunit) [C1103]; 1 : 50; Sigma) overnight at 4°C. Following two phosphate-buffered saline washes, appropriate Cy2 (1 : 500)- or Cy3 (1 : 250)-labeled secondary antibodies (Dianova, Hamburg, Germany) were applied for 15 min at 37°C. Cells were then nuclear counter-stained with 4,6-diamidino-2-phenylindole (Sigma) and mounted in Mowiol (Hoechst, Frankfurt, Germany). Fluorescence was observed and recorded using a Zeiss Axioplan 2 fluorescence microscope equipped with a CCD camera and AxioVision software (Carl Zeiss, Oberkochen, Germany). All images of one culture condition were acquired with identical exposure times, no subsequent image alterations were performed. For quantification of voltage-gated calcium channels (VGCC) expression, the intensity of the immunostaining was analyzed using Image J (Free Java software provided by the National Institutes of Health, Bethesda, Maryland, USA). Oval region of interests (ROI) comprising the cell soma were defined for randomly chosen dopaminergic and non-dopaminergic neurons and staining intensity was captured as mean gray value of the ROI. Staining intensities were normalized to be able to compare values of several cultures and then subjected to statistical analysis.

### Transition metal treatment

At DIV2, MDN cultures were exposed to different transition metals for 3 h before being cryo fixed. To this means, cultures were supplemented with FeCl_2_ (final concentration 50 μM) or FeCl_3_ (50 μM) or MnCl_2_ (500 μM) or MnIII-pyrophosphate (50 μM, all Sigma-Aldrich).

### Sample preparation for X-ray imaging

The cells grown on the silicon nitride membrane were briefly washed in 180 mM ammonium acetate buffer (pH 7.4) to eliminate the culture medium. This buffer was chosen to avoid osmotic shock and to remove the inorganic ions. Samples were quickly blotted with fine filter paper and immediately rapidly plunged in liquid ethane cooled with liquid nitrogen to up to −196°C and then stored under liquid nitrogen until imaging.

For some experiments, freeze-dried samples were used, as indicated below. Samples were shock frozen as described previously and directly afterward freeze dried at maximum −100°C for 2 days, and over the next 2 days the temperature was increased slowly to 20°C, and samples were stored in a desiccator over silica gel at 20°C.

### Fluorescence light microscopy *in vivo*

Immediately prior to sample freezing, all cultures were completely imaged to create a localization map of the cells on the silicon nitride membrane and to identify GFP-positive DAergic neurons for subsequent X-ray imaging. Photomicrographs were taken using an inverted fluorescence microscope (Axiovert; Zeiss), while the culture was kept in a heated (37°C) imaging chamber under controlled atmosphere (5% CO_2_). For each visual field, images at 10 × magnification were taken using phase contrast and EGFP filter.

### X-ray fluorescence microscopy in cryo conditions

The spectromicroscopy experiments were carried out at the undulator beamline ID21 of the European Synchrotron Radiation Facility (ESRF) in Grenoble. ID21 is a windowless ultra high vacuum beamline—continuous with the storage ring vacuum, dedicated for X-ray microscopy and spectromicroscopy (Cotte 2007). The recently developed cryogenic sample environment at beamline ID21 at ESRF allowed cryo measurements at liquid N_2_ temperature, i.e. to perform direct measurements in the cells without freeze drying.

The experiment was carried out at photon energies E = 6.6 keV for Mn-treated samples and 7.2 keV for Fe-treated samples, and the control samples were imaged on both excitation energies. A Kirkpatrick–Baez (KB) mirror system was used as focusing optics for this experiment, providing a spot size of 0.35 × 0.9 μm (V × H) with a flux of 6 × 10^10^ photons/s/Si(111) bandwidth.

The sample is aligned in the focal plane of the KB and raster scanned in the microbeam to collect 2D images. Silicon photodiodes are used for transmission measurements. Fluorescence photons are collected by a high-purity germanium detector (HpGe, Princeton Gamma-Tech) and/or a Silicon Drift Diode (SDD, XFLASH 2001, Rőntec, Berlin, Germany). Transmission signal as well as full fluorescence spectra can be recorded for each pixel of the map.

A two-dimensional analysis of elemental distribution in samples of MDN cultures was performed, with areas of up to 100 × 100 μm or smaller and mapped with steps of 300–500 nm. The measurement time (dwell time per pixel) was in the range of 150–800 ms per pixel. All measurements were performed in fluorescence mode and normalized. Summarized spectra of each measurement were presented in addition.

A photodiode, monitoring the fluorescence signal from a thin Ti foil inserted in the beam, provided a measurement proportional to the incident beam flux I0, upstream the sample, the ‘iodet’ counter. To normalize the XRF maps, the deconvolved elemental maps (obtained from PyMca fitting) were divided by the iodet map.

The areas of interest were selected by means of an on-line optical microscope, normal to the sample face. A digital camera was used for observation in transmission and sample alignment. Sample areas were selected before the raster scans in the X-ray microscope without the need of sample transfer out of the chamber. Previously obtained fluorescence microscopy images correlating with the localization maps were used to select the cells for the X-ray raster scan.

Besides element distribution by XRF, the scanning transmission X-ray microscope at ID21 permitted spatially resolved X-ray absorption spectroscopy (XAS) at cellular and subcellular levels. This instruments allows X-ray absorption near edge structure (XANES) spectroscopy, and provides information primarily about geometry and oxidation state at the K-absorption edge of manganese at E = 6539 eV and iron at 7112 eV, combined with high spatial resolution. This ability gave the unique opportunity to examine Fe and Mn in neural cells with both high spatial and high spectral resolution. The fluorescence detector was used to obtain maps of the trace metals distribution within these cells. These maps resolved metal-rich areas. XANES spectra have been taken to identify the oxidation state at these spots. Taking images at the peak energies of the respective resonance lines from these compounds will yield maps of its distribution within the studied cellular structures. As references, spectra of MnCl_2,_ Mn(III) pyrophosphate, as well as FeCl_2_, FeCl_3_, were taken at 6539 and 7112 keV, respectively. The energy range for Mn spectra was in the range from 6500 to 6700 eV, and for Fe spectra usually from 7140 to 7280 eV. The step size was 0.25 eV.

X-ray absorption near edge structure spectra were acquired in fluorescence mode and normalized by the incident beam flux (I0). Then, a XANES normalization procedure was applied: first, a linear background was fitted in the pre-edge region and subtracted from the spectrum. Second, the edge jump was normalized to unity. The micro-XANES spectra acquired on the samples were then fitted with a linear combination of XANES spectra from known standard compounds to assess the proportions of the different Fe forms.

### Data analysis and statistics

All samples were prepared at least in triplicate, in several independent experiments. For image and spectra analysis, we used PyMca and XOP software (ESRF, Grenoble, France), free available software for X-ray fluorescence analysis developed at the ESRF. The software allows interactive as well as batch processing of large data sets and is particularly well suited for X-ray imaging. The algorithms employed are described in detail elsewhere (Cotte 2007). Differences between groups were considered statistically significant according to a one-way anova and *post hoc* Dunnett test or the Mann–Whitney *U*-Test. Significances were indicated with *p < 0.05; **p < 0.01; ***p < 0.001, unless otherwise stated.

## Results

### Identification of DAergic neurons in the MDN culture

To clearly identify DAergic neurons in the mixed MDN culture, localization maps of all imaged silicon nitride membranes were taken prior to cryoplunging. DAergic neurons were easily identified as GFP-positive cells by fluorescence microscopy (Figure S1). These maps served later for unequivocal identification of neurons for the X-ray analysis.

### Effects of transition metals on DAergic neuron survival

Treatment of MDN cultures with transition metals has previously been shown by our group to have detrimental effects on DAergic neuron survival (Lingor *et al*. 2000). In the present experiments, we attempted to achieve comparable toxicity *in vitro*, which required treatment with different concentrations of transition metal ions. To confirm similar survival rates at 3 h after application of transition metals, we counted TH-immunopositive cells. In comparison with non-treated control cultures (100%), treatment with Mn^2+^ (500 μM), Mn^3+^ (50 μM), Fe^2+^ (50 μM), or Fe^3+^ (50 μM) resulted in moderately reduced survival of DAergic neurons (74.9 ± 7.4%, 71.7 ± 12.6%, 65.9 ± 4.7%, 71.8 ± 5.4%, respectively) (Figure S2).

### Spatial and elemental information yielded by X-ray fluorescence analysis

By raster scanning the sample and mapping the fluorescence yield, the intracellular distribution of the accessible chemical elements Na, Si, P, S, Cl, K, Ca, Mn, and Fe can be determined in a single cell at a spatial resolution corresponding to the beam size.

**Fig. 1:**
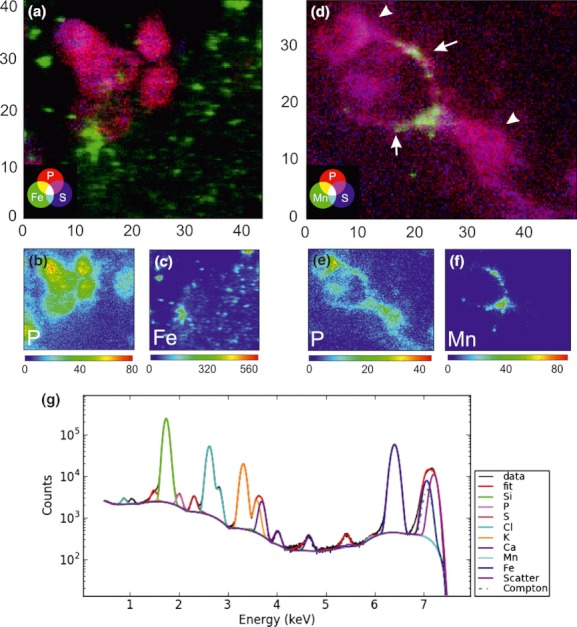
Typical X-ray fluorescence maps and summarized spectrum of freeze-dried primary dopaminergic neurons at 7.2 keV. (a) Merged maps indicating Fe (green), P (red), and S (blue) after treatment with 50 μM Fe^2+^. (b, c) Single element maps for P (b) and Fe (c) of cells shown in (a). Image size: 45 × 40 μm, pixel size 0.3 × 0.3 μm, dwell time 500 ms. (d) Merged maps indicating Mn (green), P (red), and S (blue) after treatment with 500 μM Mn^2+^. Arrows indicate neurites, arrow heads indicate the cell somata. (e, f) Single element maps for P (e) and Mn (f) of cells shown in (d). Image size: 49 × 40 μm, pixel size 0.3 × 0.3 μm, dwell time 500 ms. Dynamic color scales indicate number of normalized counts. (g) Typical cumulative XR-fluorescence spectrum corresponding to the measurement shown in (a–c). The following peaks can be distinguished: data (black), fit (red), Si (green), P (magenta), S (brown), Cl (light blue), K (orange), Ca (purple), Mn (cyan), Fe (blue), Scatter (pink), Compton (dark green dotted).

For our first experiments and to establish the imaging technique freeze-dried samples of cultures treated with Fe or Mn were used. Cell bodies as well as neurites could be clearly distinguished by their characteristic content of P and S and their characteristic morphology (Fig. 1a and d). Trace metals of interest, such as Fe (Fig. 1a and c) and Mn (Fig. 1d and f) could be mapped in relation to cellular structures. For example, trace metal distribution was observed in extracellular (Fig. 1a) or intracellular localization, with a distinction between somatic or neuritic distribution (Fig. 1d). The typical summarized fluorescence spectrum of a whole imaged sample is shown in Fig. 1g.

### Distribution of Mn and Fe in vehicle-treated control MDN cultures

The novel improved cryo stage and transfer system at ID21 offered the opportunity to map the elemental distribution in frozen-hydrated cells. Therefore, to conserve the physiological elemental distributions in their original oxidative state and with a possibility to analyze their oxidative states, all further measurements were performed in cryo-fixed (frozen-hydrated) samples. This setup allowed the analysis of larger sample numbers to obtain statistically relevant information.

**Fig. 2:**
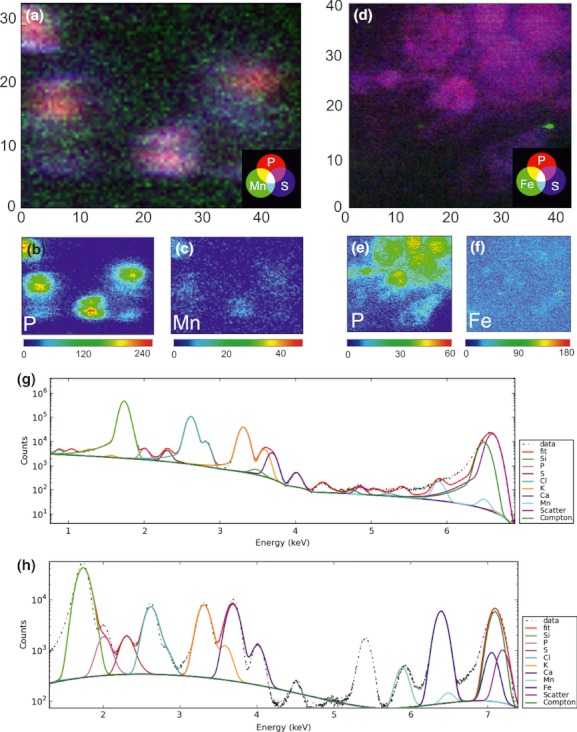
Typical XRF maps and summarized spectrum of vehicle-treated midbrain dopaminergic neuron (MDN) at 6.6 keV (a–c, g) and 7.2 keV (d–f, h). (a) Merged maps of cryo-fixed MDN indicating Mn (green), P (red), and S (blue). (b, c) Single element maps for P (b) and Mn (c) of cells shown in (a). Image size: 46 × 34 μm, pixel size 0.5 × 0.5 μm, dwell time 800 ms. (d) Merged maps of freeze-dried MDN indicating Fe (green), P (red), and S (blue). (e, f) Single element maps for P (e) and Fe (f). Image size 45 × 40 μm, pixel size 0.3 × 0.3 μm, dwell time 500 ms. Dynamic color scales indicate number of normalized counts. (g, h) Typical cumulative XR-fluorescence spectrum corresponding to the measurements shown in (a–c or d–f), respectively. The following peaks can be distinguished: data (black dotted), fit (red), Si (green), P (magenta), S (brown), Cl (light blue), K (orange), Ca (purple), Mn (cyan), Scatter (pink), Compton (dark green dotted).

To make a reliable semiquantitative statement on transition metal distribution in our treated cultures, we first imaged untreated control-MDN cultures. A very low basal content of Mn and Fe was detected in all analyzed neurons, which was homogeneously distributed in the soma (Fig. 2). The distribution of Na, Ca, Cl, S, and K was also mapped (Figure S3).

### Distribution of Fe in MDN cultures after treatment with Fe^2+^ and Fe^3+^

**Fig. 3:**
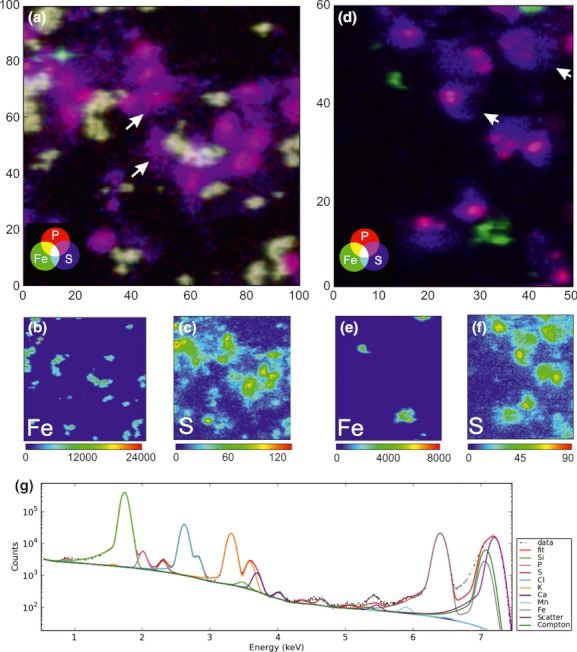
Typical XRF maps and summarized spectrum of midbrain dopaminergic neuron (MDN) treated with 50 μM Fe^2+^ (a–c) and 50 μM Fe^3+^ (d–f) at 7.2 keV. (a) Merged maps of cryo-fixed MDN indicating Fe (green), P (red), and S (blue). (b, c) Single element maps for Fe (b) and S (c) of cells shown in (a). Image size 100 × 100 μm, pixel size 1 × 1 μm, dwell time 500 ms. (d) Merged maps of cryo-fixed MDN indicating Fe (green), P (red), and S (blue). (e, f) Single element maps for Fe (e) and S (f) of cells shown in (d). Image size 50 × 60 μm, pixel size 0.5 × 0.5 μm, dwell time 600 ms. Dynamic color scales indicate number of normalized counts. (g) Typical cumulative XR-fluorescence spectrum corresponding to the measurement shown in (a–c). The following peaks can be distinguished: data (black dotted), fit (red), Si (green), P (magenta), S (brown), Cl (light blue), K (orange), Ca (purple), Mn (cyan), Fe (gray), Scatter (pink), Compton (dark green).

Cultures were exposed to 100 μM FeCl_2_ or FeCl_3_ for 3 h before cryoplunging. From a total number of 133 observed neurons treated with Fe^2+^, eight cells were identified as DAergic neurons. A strong signal for Fe colocalizing with the signal for P (indicating colocalization with a cell) was detectable with 62.5% of DAergic neurons (5/8) and with 52% of non-DAergic neurons (65/125). The distribution of Fe seemed to colocalize mostly to the vicinity of or in neuronal somata, while localization in neurites was not observed. The detailed analysis of the maps for P, S, and Fe suggested, however, that the majority of the Fe signal morphologically most likely corresponds to extracellular accumulations, not to an intracellular localization of Fe, even though the 2D analysis performed here is not able to unequivocally resolve this question. There was no marked difference of Fe distribution in regard to DAergic and non-DAergic neurons (Fig. 3a–c).

After treatment of cultures with Fe^3^+, no clear intracellular distribution of Fe was observed. In contrast to the Fe^2+^-treated cultures, most of the Fe signal was of clear extracellular localization, which could be verified by colocalization with the signal for P and S. No significant difference in Fe accumulation between DAergic and non-DAergic neurons could be observed (Fig. 3d–f). Maps for P, K, Ca, and Cl were also obtained (Figure S4).

### Mn distribution in MDN cultures after treatment with Mn^2+^ and Mn^3+^

**Fig. 4:**
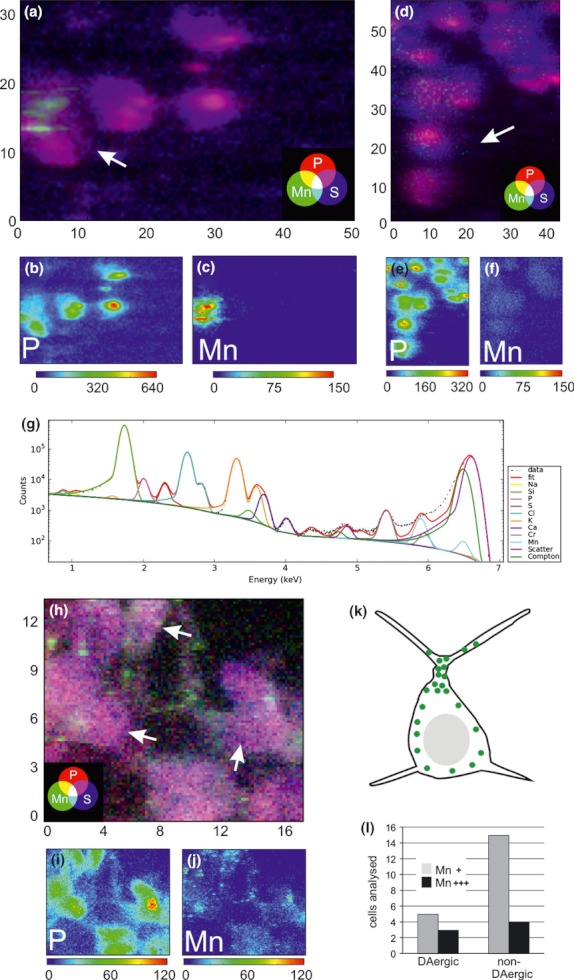
Typical XRF maps and summarized spectrum of midbrain dopaminergic neuron treated with 500 μM Mn^2+^ (a–c, h–j) and 50 μM Mn^3+^ (d–f) at 6.6 keV. (a) Merged maps of cryo-fixed cultures indicating Mn (green), P (red) and S (blue). (b, c) Single element maps for P (b) and Mn (c) of cells shown in (a). Image size 40 × 32.5 μm, pixel size 0.5 × 0.5 μm, dwell time 800 ms. (d) Merged maps of cryo-fixed cultures indicating Mn (green), P (red), and S (blue). (e, f) Single element maps for P (e) and Mn (f) of cells shown in (d). Image size 45 × 60 μm, pixel size 0.5 × 0.5 μm, dwell time 800 ms. Dynamic color scales indicate number of normalized counts. (g) Typical cumulative XR-fluorescence spectrum corresponding to measurement shown in **(**d–f). The following peaks can be distinguished: data (black), fit (red), Na (yellow), Si (green), P (magenta), S (brown), Cl (light blue), K (orange), Ca (purple), Chromium (gray), Mn (cyan), Scatter (pink), Compton (dark green). (h) Merged maps of freeze-dried cultures indicating Mn (green), P (red) and S (blue). Single element maps for P (i) and Mn (j) of cells shown in (h). Image size 17 × 13 μm, pixel size 0.5 × 0.5 μm, dwell time 800 ms. Dynamic color scales indicate number of normalized counts. Arrows in (a, d, and h) indicate dopaminergic neurons. (k) Schematic representation of typical manganese distribution (green dots) found in Mn^2+^-treated midbrain dopaminergic neuron. (l) Quantification of DAergic and non-DAergic neurons with strong (Mn+++) and weak (Mn+) fluorescence for Mn.

Cultures were exposed to 500 μM MnCl_2_ or 50 μM MnIII-pyrophosphate for 3 h before cryoplunging. After treatment with Mn^2+^, we found that Mn is mostly localized intracellularly and with a preference for DAergic neurons. Here, we found that 27% of non-DAergic neurons (4/15) and 60% of DAergic neurons (3/5) show a strong signal for Mn (Fig. 4a–c, l). Closer examination of the morphology in freeze-dried samples suggested a mostly cytoplasmic or paranuclear localization of Mn. Areas with high Mn content were likely to correspond to the perinuclear cytoplasm, axon hillock, or neurites (Fig. 4h–j, k).

After treatment with Mn^3+^, a very weak, homogeneous, and mostly cytoplasmic Mn signal was detectable in a majority of the imaged non-DAergic neurons, which was similar in the DAergic neurons (Fig. 4d–f). The lower intensity of Mn in the Mn^3+^-treated cultures was likely to be because of the lower concentration of the Mn in the treatment solution. In addition, maps for S, K, Ca, and Cl were recorded (Figure S5).

### X-ray absorption near edge structure analysis of Fe in dopaminergic neurons of MDN cultures

**Fig. 5:**
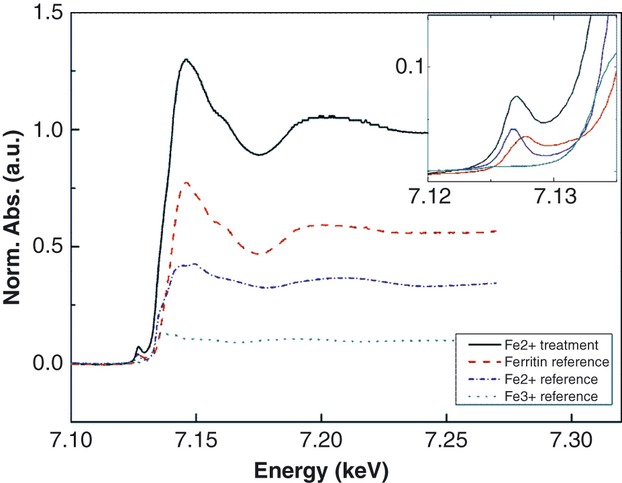
X-ray absorption near edge structure absorption spectra of a typical Fe-rich area in the cryo-fixed midbrain dopaminergic neuron culture treated for 3 h with 50 μM Fe^2+^. Absorption spectra are shown as normalized absorption in arbitrary units. Fitted spectra, black; ferritin reference, red; Fe^2+^ reference, blue; Fe^3+^ reference, green. Inset shows the pre-peaks of the respective spectra in higher magnification.

X-ray absorption near edge structure spectra were measured in the areas of highest metal signal in the cells and analyzed as a linear combination of reference spectra of Fe^2+^, Fe^3+^ (Fe_2_Cl and Fe_3_Cl, respectively) and ferritin. After Fe^2+^ treatment, we found Fe mostly to be present as Fe^3+^ (46.7 ± 8.1%) or in a ferritin-bound state (45.1 ± 6.7%), which nicely reproduced the edge position. Interestingly, Fe^2+^ contribution in the spectra was only 8.3 ± 3.1% (Fig. 5). After treatment with Fe^3+^, the accumulation of Fe in the cells was generally lower and corresponding spectra were noisier. The edge position mostly corresponds to ferritin (46.3 ± 9.1%), Fe^3+^ (19.4 ± 2.3%), and Fe^2+^ (34.3 ± 8.8%).

We also performed XANES analyses in Mn-treated cells. However, because of a very low signal, the estimation of the data was not reliable.

### Expression of voltage-gated calcium channels in the MDN culture

**Fig. 6:**
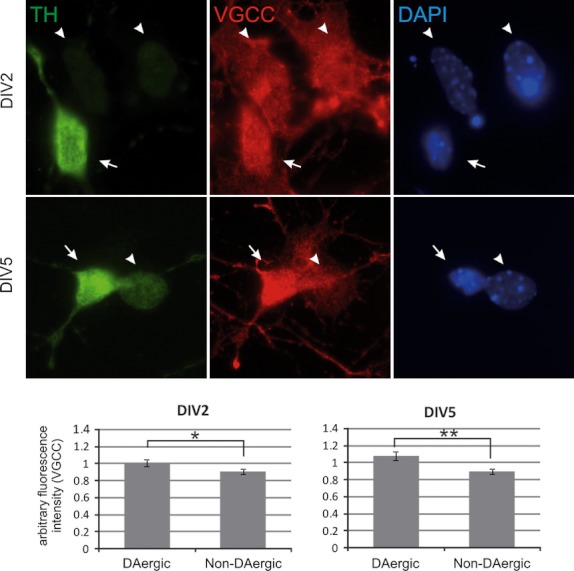
Expression of voltage-gated calcium channels in midbrain dopaminergic neuron (MDN). Representative photomicrographs of MDN after day *in vitro* 2 (DIV2) and DIV5 immunostained for tyrosine hydroxylase (TH, Cy2, green) and calcium channel α1 subunit [voltage-gated calcium channels (VGCC), Cy3, red]. Nuclear counter-stain with 4,6-diamidino-2-phenylindole (DAPI) (blue). Full arrows point at TH-positive dopaminergic neurons, arrow heads point at non-dopaminergic neurons. Diagrams demonstrate the quantification of normalized VGCC immunofluorescence in arbitrary units for MDN at DIV2 (evaluation of 24 DAergic and 70 non-DAergic cells) and DIV5 (evaluation of 23 DAergic and 69 non-DAergic cells). *p < 0.05, **p < 0.01 by Mann–Whitney *U*-test.

Our observations suggested that DAergic neurons could be more prone to incorporate manganese as compared with their neighboring non-DAergic neurons. Because calcium channels may facilitate manganese entry into cells (Crossgrove and Yokel 2005; Mertz *et al*. 1992), we considered the possibility of a differential calcium-channel expression in DAergic and non-DAergic neurons. Immunocytochemical analysis revealed that expression of VGCC is significantly higher in DAergic neurons as compared with non-DAergic neurons in the MDN culture at 2 and 5 days *in vitro* (Fig. 6).

## Discussion

Transition metals, such as manganese and iron, have been suggested to be important pathogenetic factors for a number of neurodegenerative diseases, including the most frequent neurodegenerative movement disorder, PD. In particular, metal ions have not only been implicated in mediating oxidative stress, e.g., by catalyzing the Fenton reaction, but have recently also been shown to increase aggregation of α-synuclein (Kostka 2008; Uversky *et al*. 2001). Although PD is now increasingly recognized as a system disorder with a spreading pathology (Braak *et al*. 2003), which finally affects most regions of the brain, the special vulnerability of the dopaminergic nigrostriatal projections is particularly intriguing. In this study, we aimed to correlate the localization of the transition metals iron and manganese with the transmitter phenotype of midbrain neurons and evaluate in more detail the subcellular localization of these transition metals after treatment with different oxidative states of Fe and Mn.

We used primary MDN cultures from transgenic mice expressing GFP under the TH promoter (Matsushita 2002), which allowed us to clearly identify DAergic neurons in this mixed primary neuron culture and to correlate metal distribution to the transmitter phenotype (Figure S1 and Fig. 1). The metal ion concentrations employed in this set of experiments were chosen to achieve a slight, but quantifiable effect on the survival of DAergic neurons (Figure S2), which, of course, only partially are able to mimic the chronic exposure in PD patients.

To detect transition metals, we combined the above-mentioned culture technique with synchrotron radiation XRF nanoprobe analysis, which is a multielemental analytical technique enabling the simultaneous imaging of chemical elements at trace concentrations (Carmona *et al*. 2008; Ortega *et al*. 2009). In general, in comparison with X-ray electron microanalysis, synchrotron X-ray beams possess a higher spatial resolution (100 nm vs. 1000 nm with electron probe microanalysis), higher sensitivity (0.1 μg/g vs. 100 μg/g), and the ability to operate spatially resolved compounds analysis using XAS (Bacquart 2007). Using the X-ray microscope at ID21 gave us the unique opportunity to examine Fe and Mn in neural cells with both, high spatial and high spectral resolution.

Several previous reports demonstrated measurements of transition metals, which, however, were mostly performed in freeze-dried or chemically fixed samples under conditions that permit oxido–redox processes (Ide-Ektessabi and Rabionet 2005; Szczerbowska-Boruchowska 2007). Bacquart and coworkers proved better repeatability and sensitivity with no oxidation state modification as well as minimal beam damage when cells were analyzed in a frozen-hydrated state, as compared with freeze-dried cells (Bacquart 2007). Our goal was to show the localization of metals and their relative concentrations without changing their oxidative state. This was possible only by keeping the neuronal cultures deep frozen under liquid nitrogen temperature. Rapid cryogenic cooling and subsequent measurement under cryo conditions is at the moment the best option to achieve structure and chemical preservation in the sample and protection from radiation damage (Paunesku 2006).

To make a quantitative conclusion about metal distribution in different neuron subtypes, we present here data from a large number of imaged neurons, which is in contrast to many studies presenting only single-cell data. To the best of our knowledge, this is the first report directly measuring the distribution of the transition metals iron and manganese in primary midbrain neurons with a clear differentiation between DAergic and non-DAergic neurons in culture by using XRF methods combined with cryo conditions.

In our primary culture model, colocalization of iron and neurons was observed almost exclusively in Fe^2+^-treated cultures, whereas after Fe^3+^ application iron was found mostly in the extracellular space (Fig. 3). Moderate survival impairment was, however, observed after application of iron in both oxidation states (Figure S2). Our data thus support the hypothesis that the toxic effects of the redox-active Fe^2+^ are at least partially because of internalization and possibly cytoplasmic production of free radicals. Using XANES analysis after Fe^2+^ treatment, we found Fe to accumulate within the cells mostly in oxidized Fe^3+^ or ferritin-bound form (Fig. 5). Fe^2+^ is known to act as a catalyst for redox reactions and it is oxidized to Fe^3+^, e.g., via Fenton reactions, thereby fostering the production of intracellular free radicals [reviewed in (Jomova 2010)]. Our results of the XANES analysis support the notion that this oxidation indeed occurs. Iron uptake *in vivo* occurs mainly via ferritin, but non-transferrin-bound iron, e.g., as citrate has also been shown to be taken up by neurons *in vitro* (Moos 2007). On the other hand, we found that Fe is also present in the ferritin-bound form, the main iron store within the cell. The heavy chain of ferritin possesses ferroxidase activity, that involves the conversion of iron from the ferrous (Fe^2+^) to ferric (Fe^3+^) forms (Harrison and Arosio 1996). This limits the deleterious reaction that occurs between ferrous iron and hydrogen peroxide known as the Fenton reaction that produces the highly damaging hydroxyl radical.

Conversely, the toxic effects of Fe^3+^ are most likely because of its action at the cell membrane as treatment with Fe^3+^ did not result in a detectable uptake. Interestingly, using XANES analysis, we could detect a substantial amount of Fe^2+^ after treatment with Fe^3+^, which suggests the presence of extracellular reducing agents, e.g., in the cell culture medium.

The divalent metal transporter (DMT)-1 also mediates iron uptake in DAergic neurons, and rats deficient for DMT-1 are protected against iron-mediated neurodegeneration (Salazar 2008), while over-expression leads to increased dopaminergic cell death that is further aggravated by mutant α-synuclein (Chew 2011). In PC12 cells, Fe^2+^ was also shown to enter the cell via VGCC in a competitive manner with calcium (Gaasch 2007). VGCC in midbrain DAergic neurons could thus mediate iron entry into the cell independent of the DMT-1, and our analysis suggests that DAergic neurons indeed show a higher VGCC expression in comparison with non-DAergic neurons (Fig. 6). A fraction of the neurons examined in our study were DAergic, however, we did not observe a preferential colocalization with iron in this neuronal subtype. For the treatment time of 3 h, which was used in our studies, the uptake of iron did not differ significantly in DAergic versus non-DAergic cells. In humans, iron is preferentially found in DAergic neurons containing neuromelanin (NM), and NM has been suggested to act as an iron store (Fasano *et al*. 2006). NM, which accumulates in the aging human nigral DAergic neurons (Zecca 2003), however, is not present in these mouse primary cultures because of their embryonic age. This could be one explanation why we did not observe an increased colocalization of iron and DAergic neurons in this short-term-treatment model, which only insufficiently reproduces age-related alterations of the DAergic system.

Our observations in manganese-treated cultures suggest on one hand that manganese is taken up mostly in its Mn^2+^ oxidation state, whereas Mn^3+^ uptake is one magnitude lower, and that DAergic neurons show a more prominent uptake than non-DAergic neurons (Fig. 4). Moreover, manganese seems to accumulate in the apical region of the neuronal soma, possibly the axon hillock, as well as in neurites (Fig. 4). Carmona *et al*. showed that in manganese-treated PC12 cells, manganese was localized predominantly in the Golgi apparatus after exposition to 100 μM MnCl_2_, but it became more cytoplasmic when cells were treated with a higher concentration. The authors claim that the Golgi apparatus may act as a manganese storage, which becomes overloaded at toxic concentrations (Carmona *et al*. 2008). Especially low concentrations of Mn were measured after Mn^3+^ treatment, which was used in a 10-fold lower concentration to achieve the similar effect on survival as with the other treatments (Figure S2).

Unlike for iron, manganese uptake in the brain very likely does not involve the DMT-1 (Crossgrove and Yokel 2004), but manganese may be taken up by other transporters or via calcium channels such as VGCC (Crossgrove and Yokel 2005; Mertz *et al*. 1992). Our data suggested that the DAergic subpopulation might be more prone to manganese toxicity as a result of increased manganese uptake after Mn^2+^ incubation. The immunohistochemical analysis of MDN at 2 and 5 days in culture revealed that there is a significant difference in VGCC expression between DAergic and non-DAergic neurons, such that DAergic neurons show a stronger VGCC expression (Fig. 6). While this may not be the only factor, stronger VGCC expression could be at least partially responsible for increased manganese uptake in DAergic neurons and thus explain their selective vulnerability toward this transition metal.

Imaging studies in non-human primates suggest that manganese exposure markedly reduces striatal dopamine release and results in a degeneration and gliosis in the globus pallidus, the nucleus subthalamicus, and the pars reticulata of the substantia nigra, whereas the pars compacta was largely unaffected (Eriksson 1987; Olanow 1996). Together, these findings argue in favor of a pre-synaptic damage of dopaminergic neurons induced by manganese resulting in altered dopamine release [reviewed in (Guilarte 2010)]. Interestingly, a recent study suggested that LRRK2, one of the most commonly mutated proteins in familial PD, may act as a manganese sensor, establishing a link between genetic and environmental factors in the pathogenesis of PD (Covy and Giasson 2010). In addition, manganese can increase the rate of α-synuclein fibrillization *in vitro* (Uversky *et al*. 2001). Recently, dopamine secreted by the neurons and not intracellular dopamine was shown to be directly involved in the generation of toxic reactive oxygen species after treatment with Mn. The extracellularly active enzyme dual-oxidase and the dopamine reuptake transporter were proposed to be the major mediators for increased oxidative stress in DAergic neurons exposed to manganese in *C. elegans* (Benedetto 2010). Thus, the increased vulnerability of DAergic neurons may be because of their specific neurotransmitter metabolism combined with preferential manganese uptake.

In summary, our data underscore the importance of single-cell analysis and correlative imaging including novel X-ray microscopy techniques for the understanding of pathophysiological processes in models of neurodegeneration. Our study sheds light to the selective trace metal distribution in primary DAergic neurons suggesting that this subpopulation is more prone to accumulate Mn intracellularly than other neurons, which could not be observed for Fe. Uptake of divalent ions was preferred and the distribution of Mn and Fe was different regarding the cellular compartments. This differential localization and distribution could contribute to selective vulnerability of DAergic neurons in Parkinson's disease and suggests trace metals as molecular targets in future therapeutic options of this neurodegenerative disease.

## Abbreviations

DMT: divalent metal transporter
GFP: green fluorescent protein
MDN: midbrain dopaminergic neuron
NM: neuromelanin
PD: Parkinson's disease
VGCC: voltage-gated calcium channels
XANES: X-ray absorption near edge structure
XRF: X-ray fluorescence

## Supporting information

**Figure S1.** Localization map of a silicon-nitride membrane demonstrating the identification of DAergic neurons.

**Figure S2.** Survival of DAergic neurons in MDN culture after trace metal supplementation.

**Figure S3.** (a-d) Single element maps for Na (a), Cl (b), K (c) and S (d) of cells shown in (fig. 2a-c).

**Figure S4.** (a-d) Single element maps for P (a), K (b), Cl (c) and Ca (d) of cells shown in (fig. 3a-c).

**Figure S5.** (a-d) Single element maps for S (a), K (b), Ca (c) and Cl (d) of cells shown in (fig. 4a-c).
