# Supplementary material for: X-ray fluorescence analysis of iron and manganese distribution in primary dopaminergic neurons
Source: J Neurochem. 2012 Dec 5;124(2):250–61. doi: 10.1111/jnc.12073 (PMC3563009; doi:10.1111/jnc.12073)
Supplement: Supplementary file 1 [file jnc0124-0250-SD1.pdf]

Supplementary Figures:

X-ray Fluorescence Analysis of Iron and Manganese Distribution in Primary Dopaminergic Neurons

Tanja Dučić[a], Elisabeth Barski[b], Murielle Salome[d], Jan C. Koch[b], Mathias Bähr[b,c], and Paul Lingor[b,c]

[a] From the HASYLAB – DESY, Notkestrasse 85, D-22607 Hamburg, Germany

[b] Dept. of Neurology, University Medicine Göttingen, R.-Koch-Str. 40, 37075 Göttingen, Germany

[c] Cluster of Excellence "Nanoscale Microscopy and Molecular Physiology of the Brain", 37075 Göttingen, Germany

[d] European Synchrotron Radiation Facility (ESRF), 6 rue Jules Horowitz, F-38043 Grenoble, France

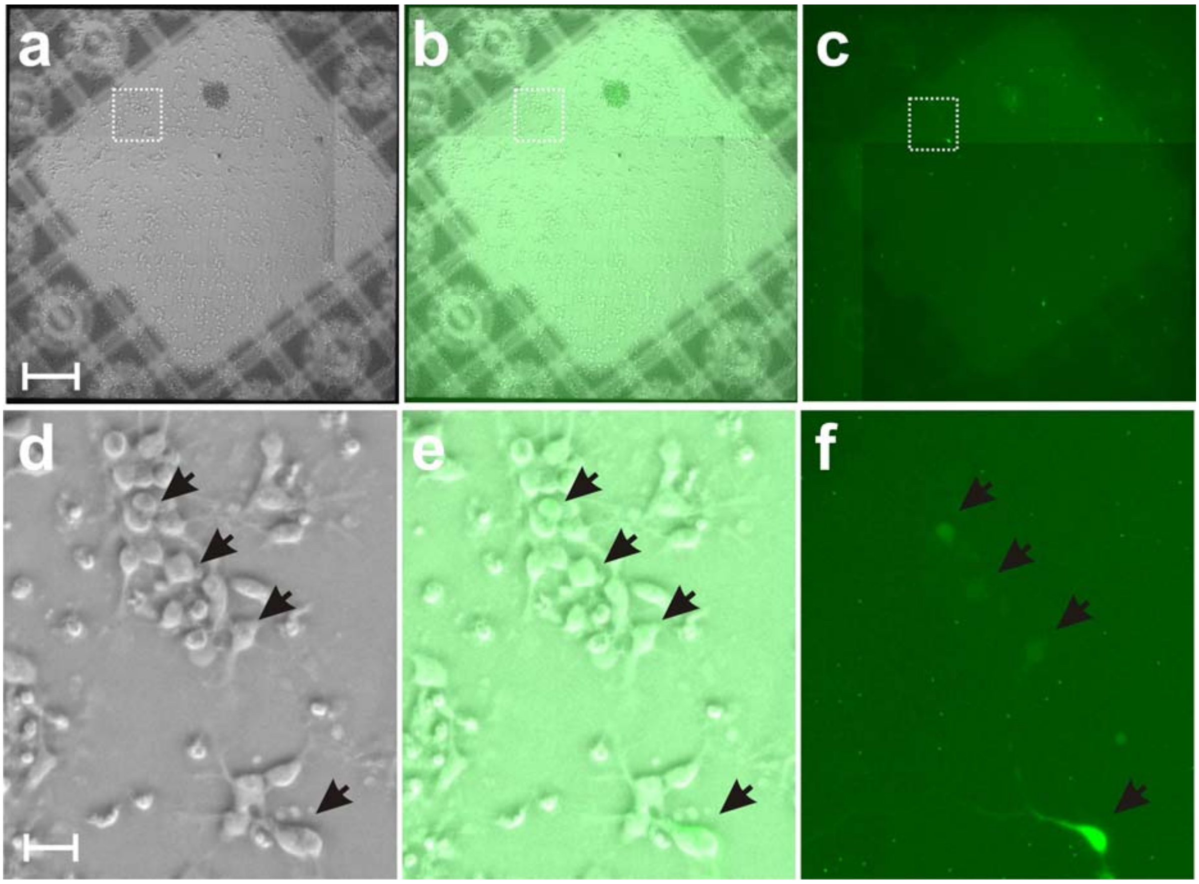

SI FIGURE 1. Localization map of a silicon-nitride membrane demonstrating the identification of DAergic neurons. (**a-c**) Composite images of the entire culture membrane at low magnification. Insets in (**a-c**) are shown in higher magnification in (**d-f**). EGFP-positive DAergic neurons can be clearly identified (arrows). (**a, d**) Phase contrast, (**c, f**) EGFP, (**b, e**) merge. Scale bar: (**a**) 200 µm, (**d**) 25 µm.

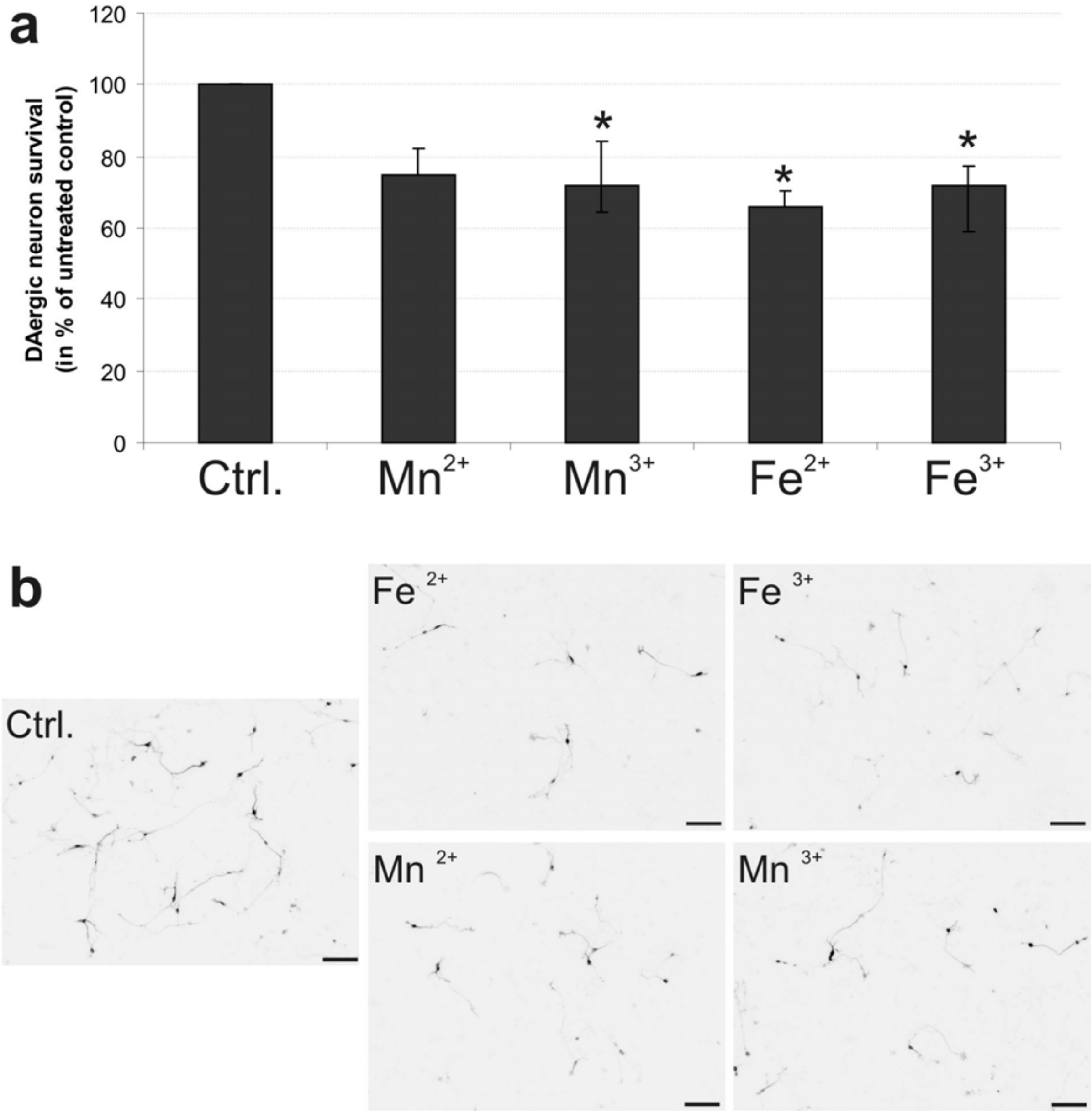

SI FIGURE 2. Survival of DAergic neurons in MDN culture after trace metal supplementation. (**a**) Cultures were treated with Mn^{2+} (500 µM), Mn^{3+} (50 µM), Fe^{2+} (50 µM) or Fe^{3+} (50 µM) for 3 hours. Percentual survival is given as mean of 4 independent experiments ± S.E.M. * $P < 0.05$ by one-way ANOVA and post hoc Dunnett test versus Control group. (**b**) Representative inverted photomicrographs showing DAergic neurons in corresponding cultures after immunocytochemical staining against tyrosine hydroxylase. Scale bars 100 µm.

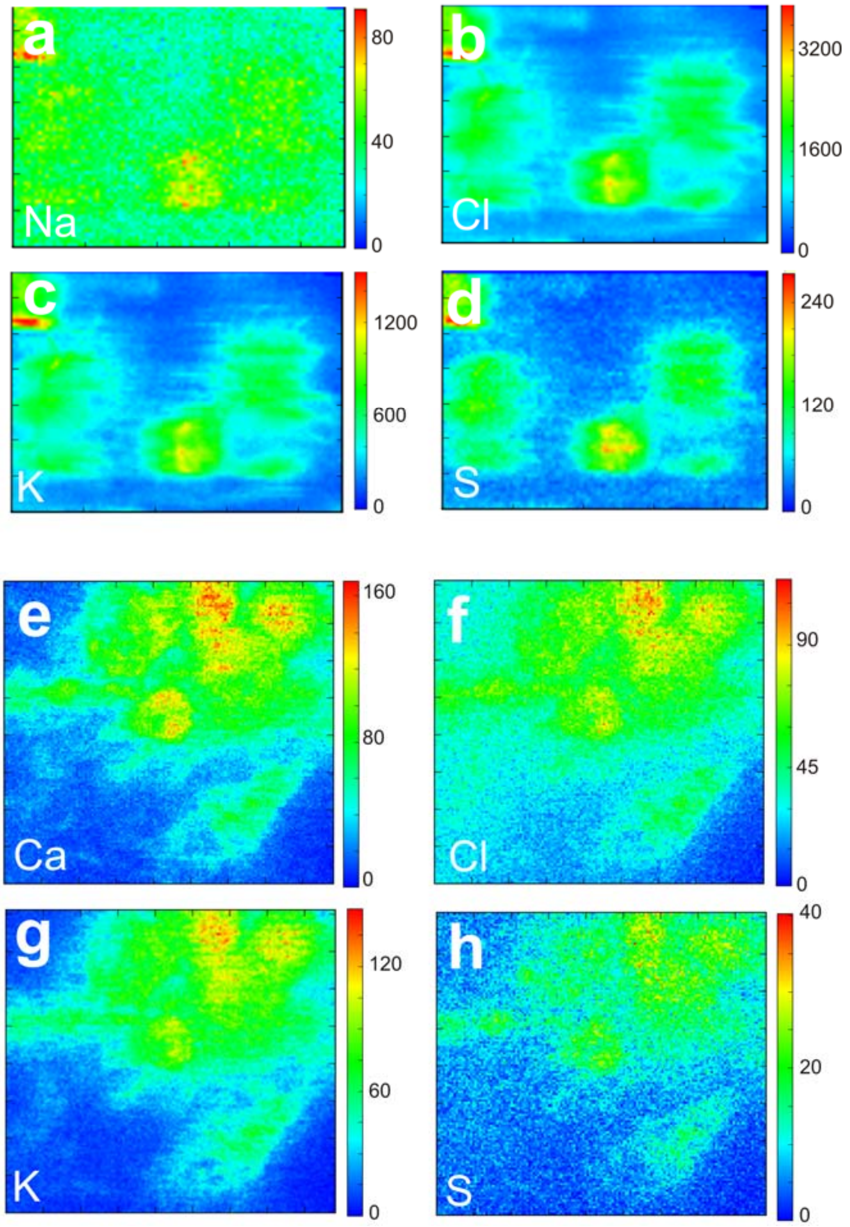

SI FIGURE 3. (**a-d**) Single element maps for Na (**a**), Cl (**b**), K (**c**) and S (**d**) of cells shown in (**Fig. 2a-c**). (**e-h**) Single element maps for Ca (**e**), Cl (**f**), K (**g**) and S (**h**) of cells shown in (**Fig. 2d-f**). Dynamic color scales indicate number of normalized counts.

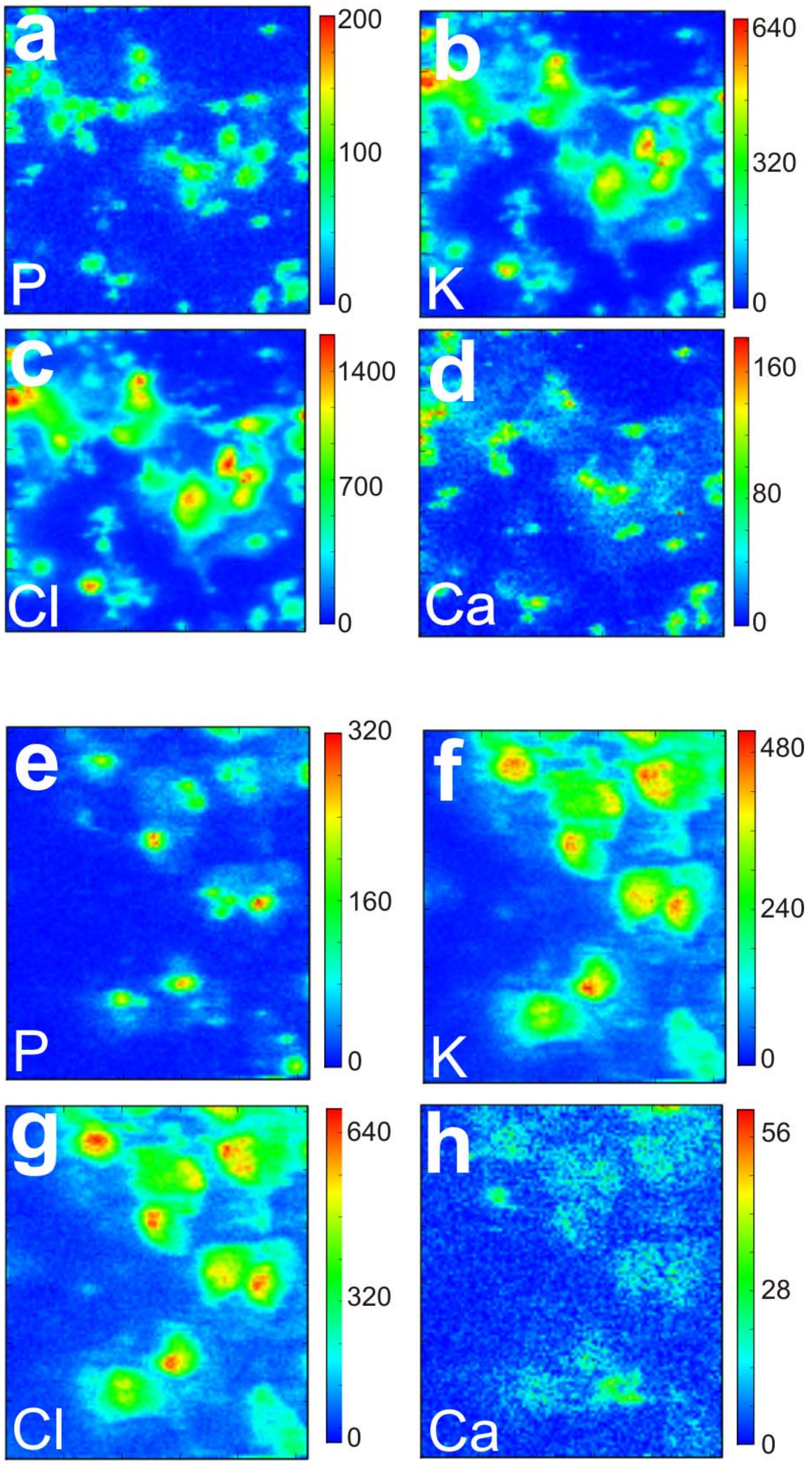

SI FIGURE 4. (**a-d**) Single element maps for P (**a**), K (**b**), Cl (**c**) and Ca (**d**) of cells shown in (**Fig. 3a-c**). (**e-h**) Single element maps for P (**e**), K (**f**), Cl (**g**) and Ca (**h**) of cells shown in (**Fig. 3d-f**). Dynamic color scales indicate number of normalized counts.

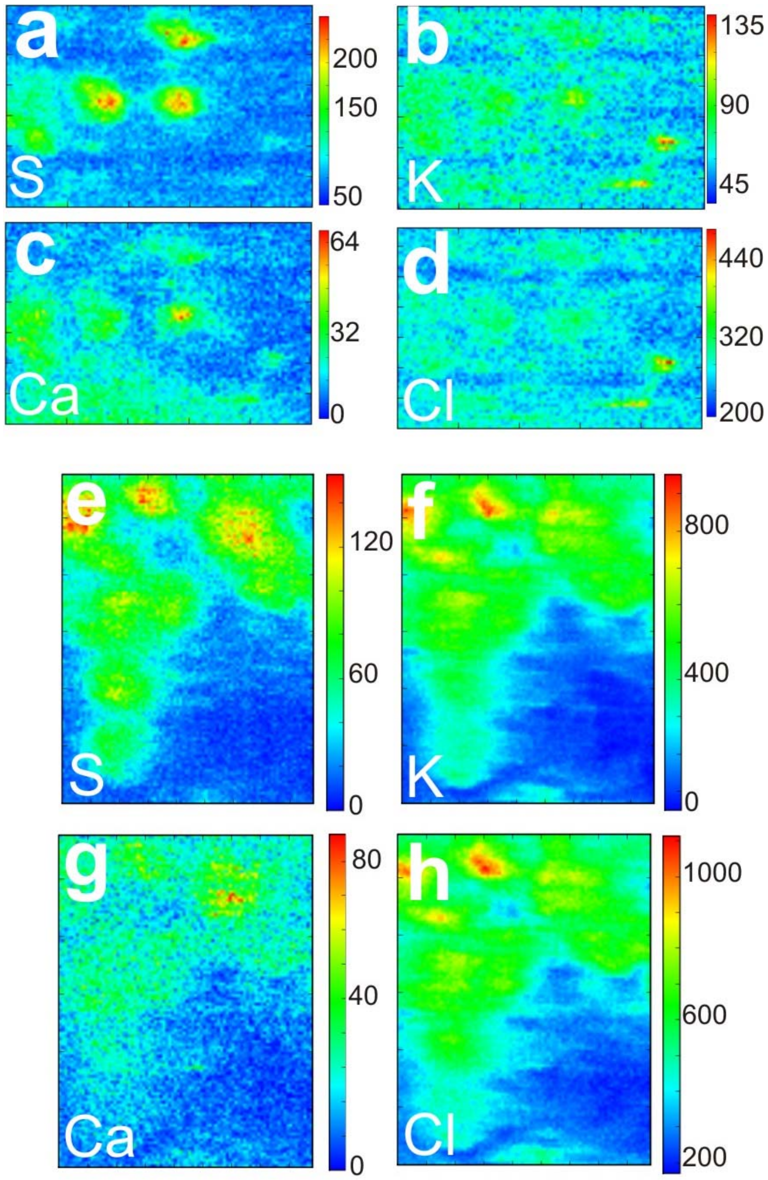

SI FIGURE 5. (**a-d**) Single element maps for S (**a**), K (**b**), Ca (**c**) and Cl (**d**) of cells shown in (**Fig. 4a-c**). (**e-h**) Single element maps for S (**e**), K (**f**), Ca (**g**) and Cl (**h**) of cells shown in (**Fig. 4d-f**). Dynamic color scales indicate number of normalized counts.
