# Supplementary figures and images for: X-ray fluorescence analysis of iron and manganese distribution in primary dopaminergic neurons
Source: J Neurochem. 2012 Dec 5;124(2):250–61. doi: 10.1111/jnc.12073 (PMC3563009; doi:10.1111/jnc.12073)

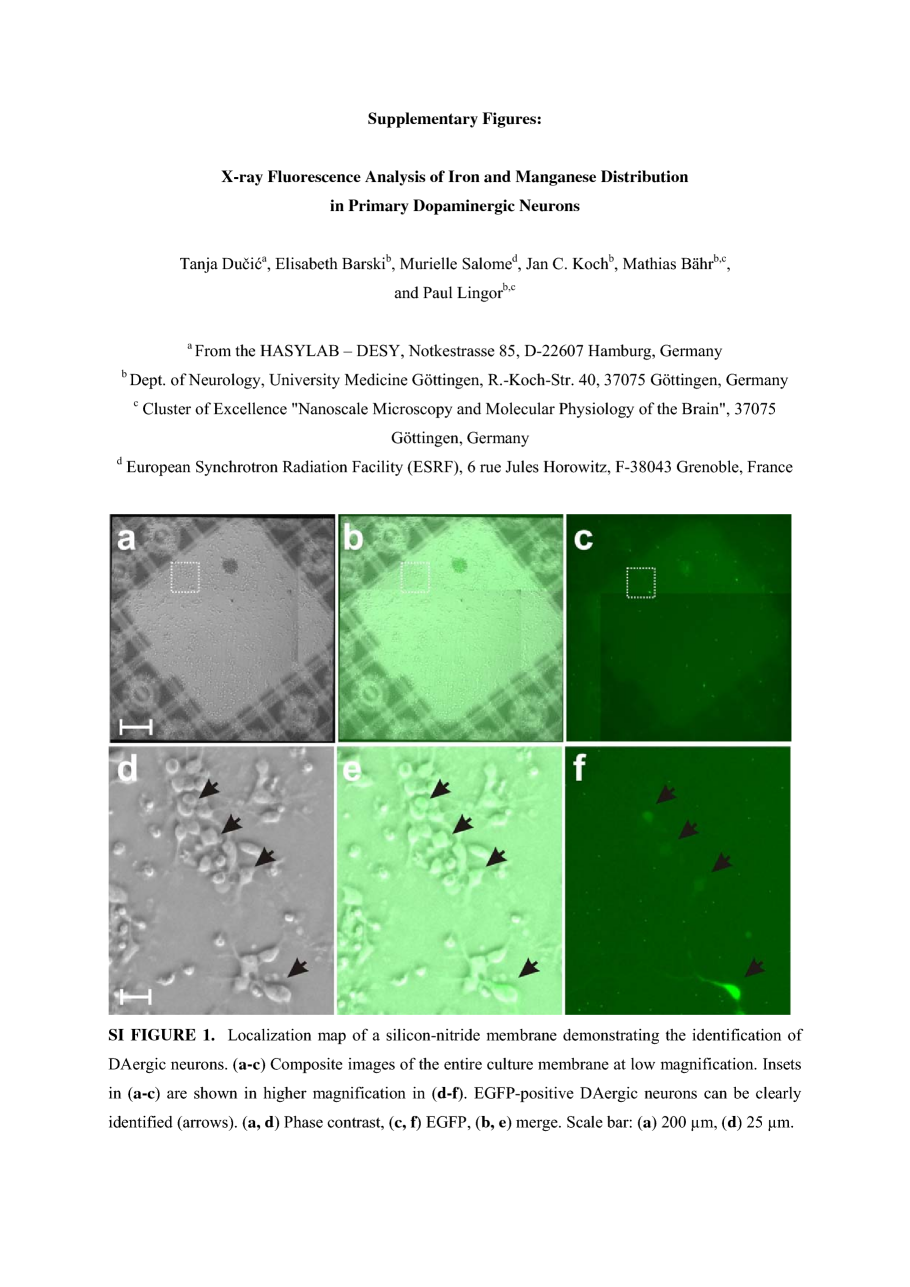

Supplement: Supplementary file 2 [file jnc0124-0250-SD2.png]
